# Treating Geriatric Sports Injury Among Pickleball Players: A Narrative Review of an Exercise Craze Among Seniors

**DOI:** 10.7759/cureus.49909

**Published:** 2023-12-04

**Authors:** Joseph Pergolizzi, Jeri Matera, Jo Ann K LeQuang

**Affiliations:** 1 Pain Medicine and Critical Care Medicine, NEMA Research, Inc., Naples, USA; 2 Pharmacology, NEMA Research, Inc., Naples, USA; 3 Pain Management, NEMA Research, Inc., Naples, USA

**Keywords:** seniors, topical pain relievers, geriatric sports injury, musculoskeletal injury, sports injury, pickleball

## Abstract

The sudden and enormous popularity of pickleball has included a surprising and large contingent of geriatric players. Similar to tennis and badminton, pickleball is a game with a short learning curve that offers low-impact cardiovascular benefits. Unlike tennis, most injuries in pickleball are sustained by older rather than younger players. In fact, pickleball-related injuries increase with increasing age. Such injuries include strains, sprains, joint pain, falls, and fractures. The most affected joints are the wrists, shoulders, knees, and ankles. Clinicians can advise their older pickleball patients on strategies and tips to minimize the risk of injury. This may be particularly important because many older individuals playing pickleball today were previously sedentary. Older people may be attracted to pickleball because it is an inclusive sport with a high socialization factor. Nevertheless, pickleball can deliver an excellent cardiovascular workout and it may be an example of a successful way to promote exercise among older people and those who resist exercise.

## Introduction and background

Pickleball is a popular American sport derived from elements of tennis and table tennis. It is played based approximately on the rules of badminton. The competitors play on a hard-surfaced court approximately 20 x 44 feet (6 x 13.4 meters), and the object is to volley a Wiffle ball back and forth over the net. With large paddles, underhand service, a non-volley zone on either side of the net, and a ball with a relatively predictable bounce, pickleball is easy for novices and older people to play, although young, fit, and very athletic players compete in fast-paced, challenging games [1]. In fact, the game was designed to discourage high-speed play and allow for all ages and abilities to participate [2]. The rapidly growing popularity of pickleball among seniors, even those who previously had been sedentary, has given the older population new recreational opportunities, while at the same time, presenting clinicians with geriatric sports injuries. Older players are the largest group of pickleball fans and also the most likely to be injured [2,3].

Invented in 1965, pickleball is an American sport created by Senator Joel Pritchard from the state of Washington and his friend, businessman William Bell. It was Bell who later introduced it to Barney McCallum, who further formalized the official game rules [2]. The game took its name from the nautical sports term “pickle boat,” a humorous and disparaging term that referred to the last boat to finish a race. There is folklore that the game was named after the Pritchard’s family dog, Pickles, but Pickles joined the family after the game was invented; the dog Pickles was named for the game, not the other way around [4].

Pickleball can be played as singles or doubles, with the latter being the more typical. The winner is the first to reach 11 or more points by at least a two-point margin. Rules can be changed to meet the needs of the players. The official rules call for two non-volley zones called “the kitchen” to run parallel to either side of the net. The kitchen truncates the court, slows down play, and can make the game easier for less athletic players. Competitive players can vote to do away with the kitchen. In fact, among aggressive pickleball players, the “dink” or landing the ball exactly in the kitchen, is considered a formidable skill.

It is easy to understand why this game has grown so quickly in popularity: it is inexpensive, quick to learn, easy to play, and suitable for players of all ages and all levels of athletic skill. Inexperienced players can quickly master enough skills to play well enough for enjoyable matches. A game lasts about 15 minutes and is often played recreationally, as a social activity, rather than a competitive level [2]. Over three million Americans are counted as pickleball players, with about one-third of them playing eight or more times a year [2]. While tennis remains more popular, pickleball is more likely to be played by older people who are more vulnerable to sports-related injuries. Sports injuries associated with pickleball are starting to be the subject of case reports in the literature. Treating sports injuries in geriatric patients is a relatively new area for many clinicians. It was the aim of this narrative review to review pickleball and its epidemiology of injuries and to offer suggestions for treating seniors with pickleball sports injuries.

Methods

In July 2023, the keyword “pickleball” was searched in the PubMed database with no delimiters and yielded 15 results. Google Scholar was also searched. Since this is a new sport, the database of Newspapers.com was also searched but no scientific content was found; however, some information from newspapers was used to describe the sport and its history. The bibliographies of key articles were also searched. This is a new topic in the medical literature.

## Review

The most comprehensive attempt to date to report and quantify pickleball injuries was published by Forrester, who described 300 types of injuries based on 19,012 cases treated in emergency departments in the United States from 2001 to 2017; 91% of these injuries were sustained by people aged 50 years or older [3]. This comprehensive report on pickleball injuries must be considered somewhat dated, as the popularity and the number of pickleball-related injuries has increased markedly from 2013 to 2017. Most of those injured in pickleball (90.1%) were ≥50 years of age. Men and women were approximately equal in terms of injuries, with men sustaining 50.4% and women sustaining 49.6% of pickleball injuries, but the type of injury varied by sex, with women suffering more fractures and men more sprains and strains. The most commonly reported injuries overall were muscle strain or sprains (28.7%), but 27.7% reported fractures, most often of a lower extremity. In 88.0% of cases of pickleball injury seen in an emergency room, patients were discharged home from the emergency room following evaluation and/or treatment [3]. It must also be considered that many mild pickleball injuries were tended at home and did not result in a visit to the emergency room; thus, these injuries represent a subset of all injuries.

It is reasonable to compare pickleball-related injuries to tennis injuries since the sports are similar. In seniors, pickleball injuries were less prevalent than tennis injuries up until about 2018, when parity was achieved [5]. Thus, seniors are roughly equally likely to be injured in pickleball as in tennis, although tennis is generally considered a more aggressive sport. For senior citizens, the most prevalent types of both pickleball and tennis injuries were those related to slipping, tripping, diving, or falling. In an evaluation of 2010 to 2019 data from the U.S. Consumer Product Safety Commission’s National Electronic Injury Surveillance System (NEISS), 85% of all pickleball injuries occurred in individuals 60 years of age or older [5]. This varies from Forrester’s report that 90% of all injuries were sustained by players over the age of 50 years, but these two reports from 2020 and 2021, respectively, relied on different datasets. Underlying these statistics is the fact that pickleball demographics are skewing markedly and rapidly to older players.

In players aged 60 years and older, non-injuries during pickleball, such as cardiovascular events, accounted for 11.1% of all pickleball cases. This can be compared to 21.5% of all tennis-related cases in that same age bracket [5]. Among seniors, the most commonly sustained pickleball-related injuries were strains and sprains (33.2%), fractures (28.1%), and contusions (10.6%). Senior men were more likely than senior women to suffer a pickleball-related strain or sprain, while women were more likely to sustain a fracture. In fact, senior women were nine times more likely than senior men to fracture a wrist while playing pickleball (Table 1) [5].

**Table 1:** Type of injuries and cases that occurred among pickleball players. Based on data from Greiner [2].

Injury	Overall frequency	Of note
Slip/trip/fall/dive	63%	The most common injury overall
Hit by an object	<5%	
Hit by the paddle	<2%	
Hit with the ball	<1%	
Collide with other players	<1%	
Heat-related illness		
Strain or sprain	33%	More common in men
Fracture	28%	More common in women
Contusions/abrasions	11%	
Internal injury	10%	
Laceration	5%	
Dislocation	<5%	
Concussion	<1%	
Hematoma	<1%	
Eye injury	<1%	

Fractures, more frequent in women than men, most often involve the wrist or leg. Retinal tears and other eye injuries, although rare, have been reported in the literature; for these reasons, protective eye gear is recommended [6,7].

Why do seniors play pickleball?

The popularity of pickleball among senior citizens has surprised observers and created some clinical challenges while, at the same time, offering geriatric citizens the many known benefits of regular exercise. Pickleball may be considered a eudaemonic pursuit, that is, an activity that brings happiness, contributes to a sense of overall fulfillment, and allows participants opportunities for obvious self-improvement. Eudaemonic efforts are not purely hedonistic or pleasurable but revolve around finding happiness in a personally meaningful context [8]. In other words, eudaemonic pursuits are not just about having a good time, they are about building a better life. It has been postulated that seniors pursuing sports activities, such as pickleball, may be acting to contribute to their own eudaemonic sense of well-being [9].

In an analysis of papers relating to pickleball, it was reported that the vast majority of pickleball players were >50 years of age, and pickleball participation resulted in improvements in a sense of personal well-being, reduction of stress and depression, and enhanced satisfaction with everyday life [4]. The inclusive nature of pickleball makes it a particularly appealing sport for older people. Physical exercise can be an important component of overall health in general and mental health in particular and can be particularly beneficial in people with mental health disorders or physical limitations that might preclude them from other forms of sports [4].

In a survey of 690 active adult pickleball players ≥ 55 years of age, the main motivations for playing pickleball were stated to be increasing fitness and socialization. Those with higher skill levels also played for the sake of competition and skill mastery [10]. A survey of 3,012 pickleball players found pickleball players tended to be more task-oriented than ego-oriented. Since mastery of new skills is particularly satisfying for task-oriented individuals, learning and improving specific skills motivated most pickleball players. Yet in this survey, players often cited competition as the main benefit of pickleball play, meaning motivation (mastery of skills) and benefits (competition) differed [11]. This finding may be important as exercise, so crucial to good health, can be difficult for patients to initiate and sustain. This finding suggests that learning new skills is an important factor in embarking on an exercise program, but over time, it is the ability to compete using those acquired skills that sustains interest and participation. In a survey of 882 Greek university students, physical exercise in general could be correlated with task orientation and those who perceived they gained skills and competence were more likely to continue exercise programs seven to 14 months later [12]. While this study was conducted among young people, it may be generalizable to older individuals.

Pickleball is played by people of all ages, from ordinary citizens to celebrities, and seniors who may feel shut out of many mainstream activities can find the socialization aspect very appealing. It is possible for a senior citizen to break out of age-bounded and family activities to meet and interact with people of all ages and backgrounds - something that is invigorating and not easy to do without an activity that has broad all-ages appeal. Some players have challenged the notion that pickleball is just an “easy sport” with minimal strategy; in fact, some have said that knowing pickleball game strategy can help even senior players compete with younger, more physically fit competitors [13].

Epidemiology of pickleball injuries in seniors

The epidemiology of pickleball injuries differs somewhat from other similar sports. In all forms of racket sports, the mean age for injury is 37 years, with most sprains and strains occurring in players aged 18 to 40 years [14]. This contrasts sharply with the mean and median ages of a player with a clinically reported pickleball injury at 66 and 68 years, respectively [5]. In tennis, injury rates tend to decrease as age increases, and 27.5% of all tennis injuries occur in those ≥ 55 years, while 65.8% occur in tennis players between the ages of 14 and 54 years [15]. In pickleball, injury rates increase as age increases; few pickleball injuries are sustained by players under the age of 50 years, and injury rates increase markedly with each decade after 50 years [5]. Of all pickleball injuries, 81.4% occurred in players between the ages of 60 and 79 years [5].

Data confirm that pickleball is a beneficial cardiovascular workout. Using wearable devices, data were collected from 22 singles and 31 doubles pickleball players (mean age = 62.1 ± 9.7 years), and it was found that over 70% of playing time was spent in moderate to vigorous zones for cardiac activity [16]. The mean heart rates were 111.6 ± 13.5 and 111.5 ± 16.2 beats per minute, respectively, representing 70.3% and 71.2% of the predicted maximum heart rate. Steps per hour were significantly greater for single players than doubles [16].

Why are seniors vulnerable to pickleball injuries?

Of serious pickleball players, defined as those who play at least eight times a year, 52% are ≥ 55 years of age and 32.7% are > 65 years [13]. This does not mean that all pickleball players are seniors; the average age of a pickleball player in 2022 was 38 years and that represents a decrease from 2020 [13]. However, injury rates are disproportionately high in older players [11].

Pickleball injuries may be more severe among older players with osteoporosis or osteopenia, turning what might be a minor fall in a younger person into a fracture. Osteoporosis likely accounts for the higher rate of fractures among older female players who have higher rates of osteoporosis and osteopenia. Many, if not most pickleball injuries, are inherently preventable but older former couch potatoes may not know the rules. Many baby boomers who spend large portions of their lives outside of sports-related activities may undervalue stretching and warm-up to prevent injury. Some sports newbies may forget to hydrate during play. Played judiciously with a holistic view of overall health and well-being, pickleball is a low-impact cardiovascular workout. Taken to the extreme, it can result in repetitive stress injuries. Many older pickleball players are retired and play pickleball for hours every day, turning this low-impact cardiovascular workout into a mechanism for repetitive stress injuries.

Older pickleball players as well as newbies to regular athletic activity need to learn the basics of sports safety: meticulous attention to warm-ups and stretching, devoting days of the week to rest with no play at all, consistent hydration, healthful eating habits, cross-training, and paying attention to the body. While pickleball may seem like an easy sport to learn and play, proper techniques, such as how to hold the paddle, can minimize the risk of injury. For example, good pickleball form encourages players to hit the ball below the waist, which minimizes stress on the wrist. Pickleball players do not have to be in good physical condition to enjoy the sport, but avid players should develop a well-rounded physical exercise routine to get in shape as regular and competitive play does require fitness to prevent injuries.

Risks versus benefits

While seniors may have the highest injury rates in pickleball, the benefits of pickleball play often outweigh the risks for geriatric players. Properly pursued, pickleball is an excellent, enjoyable, low-impact cardiovascular exercise; regular cardiovascular workouts are known to improve cognition, reduce anxiety, help maintain a healthy weight, reduce cardiovascular and diabetes risks, strengthen bones, and build up muscles and endurance [17].

Actually, pickleball may be a template for a public health intervention for physical activity among previously sedentary populations. This was tested with a pickleball intervention among sedentary senior citizens living in a rural community. The 21 participants learned to play pickleball and participated in regular matches over a six-week period, during which participants improved physical and cognitive health and reported diminished pain. Participants adhered to the six-week program and reported satisfaction with their new activity. In fact, in this study, participants were eager to continue their pickleball play after the intervention concluded [18]. The combination of socialization with physical activity appears to be a good combination as is the fact that pickleball is an inclusive sport that does not make seniors feel unable to keep up.

While pickleball is not as strenuous as tennis or jogging, it can be fast-paced, which requires players to stay alert, promotes visual acuity, and can improve cognition as players strategize where to hit the ball next.

Clinical perspectives

The increased prevalence of pickleball-related injuries is largely due to an influx of new and older players, some of whom may not be familiar with precautions to take with regular physical exercise. Pickleball is itself not an inherently dangerous sport [5]. While senior players are more likely to sustain pickleball-related injuries than younger players, the benefits of pickleball must be considered when evaluating the suitability of the sport for geriatric persons. Pickleball improves physical well-being, cognition, and mood in senior citizens, even those who previously had a sedentary lifestyle [4,5]. However, clinicians must be prepared both to treat pickleball injuries and to advise their patients on how to reduce the risk of injury [19]. A table of the main types of pickleball injuries follows (Table 2).

**Table 2:** Common pickleball injuries for players of all ages.

Injury	Cause	Preventive strategy
Ankle injuries	Rapid movement, sudden changes in direction, sudden stops and starts, and lateral moves can stretch the Achilles tendon or tear the anterior talofibular ligament	Wear good running shoes, avoid extreme motions, warm up before play, and cool down, even with ice packs, afterward
Knee injuries	Rapid movements, sudden changes in direction, sudden stops and starts, and lateral moves can sprain the knee and tear the medial collateral ligament	Avoid extreme lateral or zig-zag running; if knees ache before play, use a brace during or after play. Ice down after play
Muscle aches and pains in the leg	If hamstrings are not properly warmed up, large strides or sudden moves can strain or tear quadriceps muscles	Warm up consistently for at least five to 10 minutes
Elbow injuries	Twisting the wrist when holding the paddle can cause micro-injuries or tiny tears to the forearm tendons. This can be a version of “tennis elbow”	Learn proper technique to hold paddle, return ball holding paddle below the waist. Ice down wrists after play. If wrists are sore but not injured, play with a brace
Shoulder injuries	Sudden or extreme reaching up to return the ball can cause a tear in the rotator cuff	Avoid overhead returns as much as possible
Falls	Rapid movements, sudden changes in direction, and running backward can cause a fall. Falls can be exacerbated when the hands are used to break the fall—and get injured in the process	Avoid running backward or making extreme and rapid movements on the run. It is much better to lose the point than fracture a wrist. Those with impaired balance may benefit from balance exercises

Acute injuries

The most commonly reported pickleball-related injuries are acute strains and sprains, which can often be treated conservatively. For pain after play or during rest, topical analgesics should be considered. One common sprain in both tennis and pickleball is the inversion of the ankle joint [5]. If severe enough, it may be necessary to provide crutches and reduce weight-bearing activities until the sprain heals. In some cases, a sprain may require immobilization using a brace. Compression, rest, elevation, and cold therapy are beneficial for sprains. Players should be advised to pay attention to minor aches and pains and take preventive action to avoid repetitive stress injuries or joint and muscle weakness. When aches and pain are noticed, players are advised to cool down, apply ice to the area for cold therapy, and afterward use topical analgesics rather than systemic agents such as nonsteroidal anti-inflammatory drugs (NSAIDs). NSAIDs are not recommended for long-term use in geriatric patients and may confer a risk for gastrointestinal or cardiovascular injury [20,21]. Since patients can get certain NSAIDs over the counter, clinicians should advise patients about their relative risk, particularly if individuals take these agents on a daily or near-daily basis [22].

While many pickleball-related injuries are mild and heal relatively quickly, an injury to the Achilles tendon may need weeks to resolve and even require physical therapy [5]. Strains on the Achilles tendon may be perceived at any point along the tendon, from the foot to the calf. A ruptured Achilles tendon is associated with intense and sudden pain and results in an inability to bear weight on that foot. In some cases, a ruptured Achilles tendon will require surgery [5].

Knee injuries, caused by rapid stop-start movements and extreme lateral moves, are common in pickleball and may be mild to severe [5]. Tears to the meniscus can require bracing, physical therapy, and/or surgery. Other muscles that can be strained by pickleball include the hamstrings, the hips, and the calf. Mild sprains respond well to conservative treatment, but more severe injuries may require analgesia, bracing, physical therapy, and surgery.

Wrist injuries are particularly common, often sustained when a player tries to break a fall using their hands. Pickleball players over age 65 years have more fractured wrists than the general population, and some of these injuries may require surgery [23]. The majority of wrist injuries can be appropriately treated non-surgically [23]. In a study of 204 people who sought treatment for an acute injury related to pickleball (n = 171) or paddleball (n = 33), the mean age of the patient was 65 years (range 14 to 92 years). Injuries were to the right upper extremity in 56% of cases compared to 38% on the left side and 6% with bilateral injuries. Of the entire cohort, 19.1% had surgical treatment while 80.9% could be treated nonoperatively. The nonoperative courses of therapy were steroid injections (26.1%), physical therapy (44.2%), and bracing or splitting (69.1%). For those individuals who had surgery, there was an average span of 88.5 days from initial injury to surgical intervention (range = one to 1014 days) [23]. While this study examined all injuries of the upper extremities, wrist injury (44.7%) was the most frequent presentation [23]. Of those players who had surgery, 71.8% had open reduction and internal fixation surgery for wrist fractures [23]. The most common course of treatment for nonoperative patients was splinting (69.1%) [23]. In reviewing acute injury demographics, injuries occur at roughly the same rates in men and women up to age 40, but women sustain more injuries than men from ages 40 to 79. At age 80 and beyond, parity resumes. Furthermore, injuries occur in a few players of either sex from ages 10 to 40 years, while injuries are most frequent from ages 50 to 79 years [23].

Chronic injuries

The most common chronic injuries sustained by pickleball players include plantar fasciitis and contusions of the heel due to repetitive pounding of the feet on hard playing surfaces. Acute sprains and strains can develop into chronic conditions if not properly addressed early. Likewise, an acute injury due to overextension can become chronic if not promptly and properly treated. “Pickleball wrist,” similar to tennis elbow, is a form of wrist tendinitis caused by overextension and/or repetitive use. The wrist is particularly vulnerable in pickleball because service, although not as strenuous as a tennis serve, requires considerable wrist action; moreover, the wrist is vulnerable to injury when players instinctively use their hands to break a fall.

Rotator cuff injury can occur in people who do not play any sports, but frequent pickleball play can accelerate this process. A conundrum for rotator cuff injury in pickleball is that it can occur in players who use good form and do not get “injured” directly. Shoulder-strengthening exercises may help minimize the risk of rotator cuff tears and icing the shoulders after play is another good preventive measure.

Education of senior pickleball players

**Figure 1:**
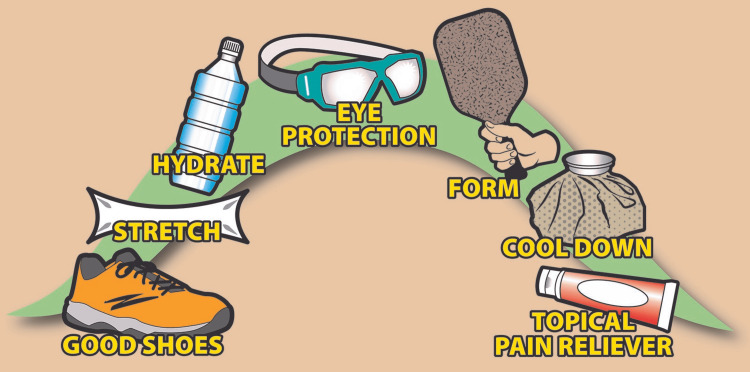
Patient education basics for preventing injury during pickleball play. This is an original image owned by the authors.

An important role of clinicians in all areas of medicine and sports medicine in particular is offering advice and counsel to patients [24]. While much pickleball-related education recapitulates the basics of injury prevention for any athletic activity, many baby boomers who have become avid pickleball players were not necessarily active in sports in school, young adulthood, or midlife. In fact, pickleball is extremely attractive to former couch potatoes who may have very limited appreciation of the basics of preventive steps to avoid the risk of sports injury. Therefore, it is good practice to offer the basics to pickleball players, at least as a starting point. See Figure 1.

To avoid chronic injury to the feet and legs, pickleball players, like runners, need to be fitted for properly fitting athletic shoes offering thick padding and support. The “toe box” or the space in the shoe between where the toes end and the shoe begins should be roomy to allow toes to slide forward a bit during running. Heel cups, orthotics, or arch supports should be utilized by those at risk for foot injuries [2,5]. Pickleball can involve running in short spurts and requires players to be able to stop short at the kitchen line, something that requires good shoe support. Pickleball players should be advised that athletes who play their sport regularly wear their shoes out over time and need to replace them regularly. Signs that shoes are wearing out include new aches and pains, particularly in the foot and ankle, uneven wear patterns on the sole, tread loss, less “bounce” in the shoe, and/or the feeling that the shoe is loose or fits poorly, even when properly tied [25].

Stretching before each match can be beneficial, but many people who are new to sports may not know this pregame step or how to stretch properly. Stretches can include a variety of bends and static positions as well as a brisk walk or jog around the court. Stretching poses should gently stretch hip adductors, quadriceps (squats), lateral bends, and gentle backbends. Lunges, arm swings, and torso twists are also included in many recommended pregame stretch routines [26]. About five or 10 minutes of stretches may be beneficial.

Older players and those new to regular physical activity should be cautioned to be realistic about their fitness level and skill set. Less fit players should be encouraged to take breaks when they feel fatigued, to get plenty of water, and to stop play if joints or muscles start to feel uncomfortable. Avid players should be reminded that good athletic programs require days off as well as days on. Players should also be advised not to “power through” minor aches and pains sustained during play but to ice them down after the game and avoid worsening the injury. Those who experience prolonged or moderate to severe soreness and muscle aches and pain should seek medical attention. In many cases, rest, an ice pack for cold therapy, and a topical pain reliever can provide at-home care. Clinicians should advise patients that braces can be readily purchased online and may provide wrist, ankle, or knee support when a joint needs some extra support. Advise patients buying wrist supports to be careful to pick the right or left wrist braces. Depending on the severity of the injury, it can be possible to resume play with a mild strain or sprain using a brace [2,5].

Overuse injuries in pickleball and tennis can sometimes be traced back more to improper form than actual overuse. Caution players to get some training or coaching as they embark on pickleball play to be sure they develop proper form and good habits. With the surge in pickleball popularity, there are many online sports sites that offer good tips and videos. For those playing pickleball at competitive levels, cross-training can be beneficial in that it will help develop muscles needed for rapid play and quick lateral motions [2,5].

Discussion

As clinicians, treating pickleball-related injuries can be a new challenge, and advising patients about pickleball injury prevention is a new addition to the counsel clinicians offer their patients. While injuries should be recognized, the overall benefits of pickleball particularly for geriatric individuals far outweigh the drawbacks. And the popularity of pickleball is something that the healthcare system should learn from.

Overall, in America, 46.4% of senior citizens participate in no leisure aerobic activities of any kind and only 26.1% stated they were regularly active [27]. The risk of having no aerobic activity at all was greater for those ≥ 65 years than those aged 50 to 64 years. Seniors in the Hispanic community (63.8%) and non-Hispanic Blacks (61.6%) had the highest rates of no aerobic exercise compared to whites of the same age brackets (43.0%) [27]. High rates of inactivity are further correlated with lower educational levels and with higher body mass index [27]. In fact, more than half of those with a BMI ≥ 30 (obese) had no leisure time aerobic activity at all [27]. These alarming statistics may explain the rapid increase in chronic diseases in our older population. In fact, recent expert consensus guidelines from the International Exercise Recommendations in Older Adults (ICFSR) advocate that physical activity be viewed as both a preventative medicine and a therapeutic agent for senior citizens [28]. A sedentary lifestyle and old age have given rise to sarcopenic obesity, a condition in which an individual has both low muscle mass and a high volume of adipose tissue [29]. Sarcopenia is associated with increased morbidity and mortality and has an incremental adverse effect on metabolic impairment, functional deficits, and many diseases, such as type 2 diabetes and cancer [30]. Thus, pickleball may be an important therapeutic option to combat sedentariness, obesity, and sarcopenia in seniors. The benefits of regular physical activity are almost too numerous to list, and there are less-often mentioned advantages that are particularly relevant to seniors: regular exercise can improve balance, boost stamina, and reduce the chance of falling in seniors while enhancing cognition, regulating mood, and delaying age-related cognitive deficits [17].

Public health interventions to increase exercise frequency and intensity have been ineffective, and many public health initiatives to increase exercise are poorly or inadequately reported in the literature [31]. Regardless of public health reporting or lack of it, it must be assumed that public health has not effectively intervened in improving exercise in America by virtue of the sedentary nature of the population. Part of the reason for this failure, besides a willingness to confront the failure, is that exercise and other health interventions are far more complicated than they seem on the surface.

It must be recognized that people have many reasons - and sometimes very good reasons - to not want to exercise. Exercise takes time, it is uncomfortable, there can be expenses involved with getting the proper equipment, and there may be a learning curve. People who are overweight or obese, out of shape, older, or just not very familiar with exercise culture can feel awkward and self-conscious venturing out in public to exercise. Rightly or wrongly, many senior citizens feel self-conscious and out of place in gyms or exercise classes, leading to self-exclusionary behavior [32]. The problem can be compounded for seniors who may experience social exclusion from other mainstream activities because of their age, appearance, or age-related limitations [33]. Older individuals have many good reasons not to exercise.

So why has pickleball been so popular among formerly sedentary seniors and how can we better learn from it to promote other exercise interventions for older people? Unlike solo gym routines or online exercise classes, pickleball is an inherently social game that involves face-to-face interactions with other people. People play pickleball with each other. Seniors in large numbers are playing pickleball, so there is less awkwardness for older individuals to play the game. The nature of the game is such that both old and young can not only play the game, but they can play fairly against each other. The age gap virtually evaporates. Furthermore, pickleball provides older players the opportunity to get to know and interact with younger people, something society does not always facilitate. The strong social aspect of pickleball is missing in other exercise regimens. This idea of getting out and interacting with real people playing a game has an innate and strong appeal that should not be dismissed when designing other exercise interventions.

Second, unlike golf or tennis, pickleball has a short learning curve and is inexpensive to play. Novices can generally gain skills sufficient to play passably well in a very short time, although the sport does offer more competitive players the chance to fine-tune their skills over time. Thus, players get the satisfaction of playing reasonably well in a short time. For seniors, this means not being outclassed right from the start in more competitive sports such as tennis, soccer, or marathon running. Furthermore, the skills that can be honed over time with pickleball are accessible to seniors. Among marathon runners, for example, there is an age barrier because all things being equal, a 60-year-old cannot compete fairly against a 20-year-old, no matter how much the older person trains. That barrier is not present in pickleball or it is at least not a brick wall.

Third, pickleball players enjoy their workout. Pickleball players do not even consider their time on the court to be exercise or a fitness routine. They are not working out, they are playing. It is a time they look forward to and a time they enjoy. It is exercise, but it is exercise disguised as fun. Many other fitness regimens emphasize work, effort, sweat, and discipline. Pickleball, from its silly name to the funny-looking modified courts, is more fun than exercise.

For these reasons, clinicians should support their local pickleball leagues and courts and encourage people to take up the sport. This includes even senior citizens and sedentary individuals. However, it is important to provide proper medical and sports-medical advice to these new and old players to minimize their risk of injury. See Figure 1.

## Conclusions

The surprising boon in pickleball and its popularity among senior citizens has increased the rate of pickleball-related injuries seen in clinical practice. Clinicians should offer treatment for these injuries, which are far more common in older than younger players, and provide advice to pickleball players on ways to minimize their risk of injury. The benefits of pickleball, particularly for geriatric players, far outweigh the risks and pickleball may be an important cardiovascular workout program that seniors ardently embrace rather than reject.
